# Evidence that spatial scale and environment factors explain grassland community assembly in woodland–grassland ecotones

**DOI:** 10.1002/ece3.11644

**Published:** 2024-07-03

**Authors:** Cheng Zheng, Haijing Shi, Jiaqi Wei, Mengying Cui, Ziqi Lin, Yuan Gao, Liuhuan Yuan, Zhongming Wen

**Affiliations:** ^1^ College of Grassland Agriculture Northwest A&F University Yangling Shaanxi People's Republic of China; ^2^ Institute of Soil and Water Conservation Chinese Academy of Sciences and Ministry of Water Resources Yangling Shaanxi People's Republic of China; ^3^ Institute of Soil and Water Conservation Northwest A&F University Yangling Shaanxi People's Republic of China

**Keywords:** community assembly, environmental gradient, functional trait, habitat filtering, limiting similarity, spatial partitioning

## Abstract

How communities of living organisms assemble has long been a central question in ecology. The impact of habitat filtering and limiting similarity on plant community structures is well known, as both processes are influenced by individual responses to environmental fluctuations. Yet, the precise identifications and quantifications of the potential abiotic and biotic factors that shape community structures at a fine scale remains a challenge. Here, we applied null model approaches to assess the importance of habitat filtering and limiting similarity at two spatial scales. We used 63 natural vegetation plots, each measuring 5 × 5 m, with three nested subplots measuring 1 × 1 m, from the 2021 field survey, to examine the alpha diversity as well as beta diversity of plots and subplots. Linear mixed‐effects models were employed to determine the impact of environmental variables on assembly rules. Our results demonstrate that habitat filtering is the dominant assembly rules at both the plot and subplot levels, although limiting similarity assumes stronger at the subplot level. Plot‐level limiting similarity exhibited a positive association with fine‐scale partitioning, suggesting that trait divergence originated from a combination of limiting similarity and spatial partitioning. Our findings also reveal that the community assembly varies more strongly with the mean annual temperature gradient than the mean annual precipitation. This investigation provides a pertinent illustration of non‐random assembly rules from spatial scale and environmental factors in plant communities in the loess hilly region. It underscores the critical influence of spatial and environmental constraints in understanding the assembly of plant communities.

## Graphical abstract

We tested the spatial dependence and environmental explanatory power of community assembly processes and clarified the importance of scale effects in community assembly by integrating multiple community assembly processes.

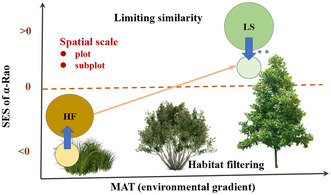

## 1 INTRODUCTION

The woodland–grassland ecotone is a complex ecosystem, which play crucial role in the carbon and nitrogen cycles, food security, and biodiversity maintenance (D'Odorico et al., 2013; Song et al., 2023). Nonetheless, these ecosystems are experiencing severe degradation due to climate change and anthropogenic pressures, with the Loess Plateau in China being particularly affected. Degradation of plants significantly lowers their capacity to provide ecological services, biodiversity, and socioeconomics (Bardgett et al., 2021). The composition and diversity of plant communities are governed by community assembly rules, which is a promising theoretical framework for the regeneration of damaged ecosystems (Gotzenberger et al., 2012). Consequently, a comprehensive understanding of these assembly rules is indispensable for informing effective restoration and management strategies for grasslands, especially in woodland–grassland ecotones.

Functional traits are the result of historical environmental shaping and species evolution, and it is evidence of species' adaptive strategies (Violle et al., 2007). Trait‐based approaches are widely employed to elucidate community assembly processes (Fu et al., 2020; Laughlin & Laughlin, 2013). The deterministic ecological process of community assembly rules involves habitat filtering and limiting similarity (Gotzenberger et al., 2012). Habitat filtering describes the convergence of trait values, where abiotic environment acts as a sieve, separating only species with certain traits to survive successfully, whereas the rest fail (Banares‐de‐Dios et al., 2020). Although habitat filtering is often associated with abiotic environments, habitats also contain biotic components, such as competitors (Kunstler et al., 2012). Limiting similarity arises from niche differentiation, where a given species must exhibit differences in resource acquisition to coexist (Barot & Gignoux, 2004). The trait diversity randomization test is a conventional method for examine the principles of community assembly (Gotzenberger et al., 2016).

Ecologists have established that biodiversity organization is nested and determined by ecological assembly rules that vary across spatial scales and environmental gradients (Jin et al., 2020; Munkemuller et al., 2014). Current research demonstrates that diversity patterns and community assembly can vary with spatial scales (Suárez‐Castro et al., 2022; Xu, Dang, Tian, Chai, et al., 2020; Yang et al., 2014). Indeed, the scale of the survey affects the patterns of community assembly, we expect assembly patterns to behave differently at different spatial scales. In fact, it has been found in some studies that community assembly at larger scales is likely to be identified as environmental filtering, specifically manifested as trait aggregation (López‐Angulo et al., 2020; Scherrer et al., 2019). Nevertheless, there is a paucity of empirical studies, and no consensus has been reached regarding the rules of community assembly in natural settings.

Spatial partitioning includes micro‐refugia created by organisms and differences in abiotic conditions. Numerous studies on habitat filtering and limiting similarity make the erroneous assumption that communities within plots are uniform (Xu, Dang, Tian, Liu, et al., 2020). The dynamics of species coexistence and community assembly are further complicated by fine‐scale environmental heterogeneity within communities. Plant species Assemblages, for instance, may result from spatial partitioning within the community. Adler et al. (2013) revealed that environmental heterogeneity influences functional trait variation, thereby facilitating species coexistence. Similarly, Tatsumi et al. (2021) suggested that dissecting the dynamic mechanisms of beta diversity could provide additional insights into the mechanisms into the spatiotemporal organization of biodiversity. To summarize, the study of community assemblage rules also requires a thorough understanding of spatial partitioning.

Assemblage rules in one habitat may not necessarily apply elsewhere (Funk, 2021). Exploring the connection between assembly rules and environmental factors can facilitate rule adaptation to other regions and addressing environment change, even though it is expected that the community assembly rules will vary with spatial scales (Banares‐de‐Dios et al., 2020; Fournier et al., 2020; Lhotsky et al., 2016; Xu et al., 2018). The stress gradient hypothesis assumption about assemblage rules is widely accepted; they propose that habitat filtering is a crucial deterministic process for low‐productivity habitats, whereas limiting similarity defines community assemblage for high‐productivity habitats (Louault et al., 2017; Louthan et al., 2015). To mitigate ecological impacts and promote biodiversity conservation, enhanced spatial partitioning strategies should be prioritized in regions experiencing significant species invasions and habitat fragmentation (Price et al., 2017; Stark et al., 2017). Hence, to understand community assembly rules more clearly, it is crucial to quantify the influence of various assembly rules across different spatial scales and environmental gradients.

In this study, we aim to improve understanding of assembly rules in woodland–grassland ecotone by focusing on spatial scale and environmental factors to reduce reliance on local pattern extrapolation. We investigated three types of habitats, four growth traits, and functional diversity of plant communities in YRB (Yanhe River basin), one of the most complex and fragile ecosystems worldwide located in the Loess Plateau of China. We established various null models utilizing two species pools to evaluate the importance of habitat filtering and limiting similarity at two different spatial scales as well as spatial partitioning within plot. Specifically, we concentrated on three questions: (1) How do species from the regional species pool become integrated into local communities? (2) Can a rule in plant communities be determined by scale? (3) What is the impact of environmental factors on the formulation of assembly rules?

## 2 MATERIALS AND METHODS

### 2.1 Study design

**FIGURE 1:**
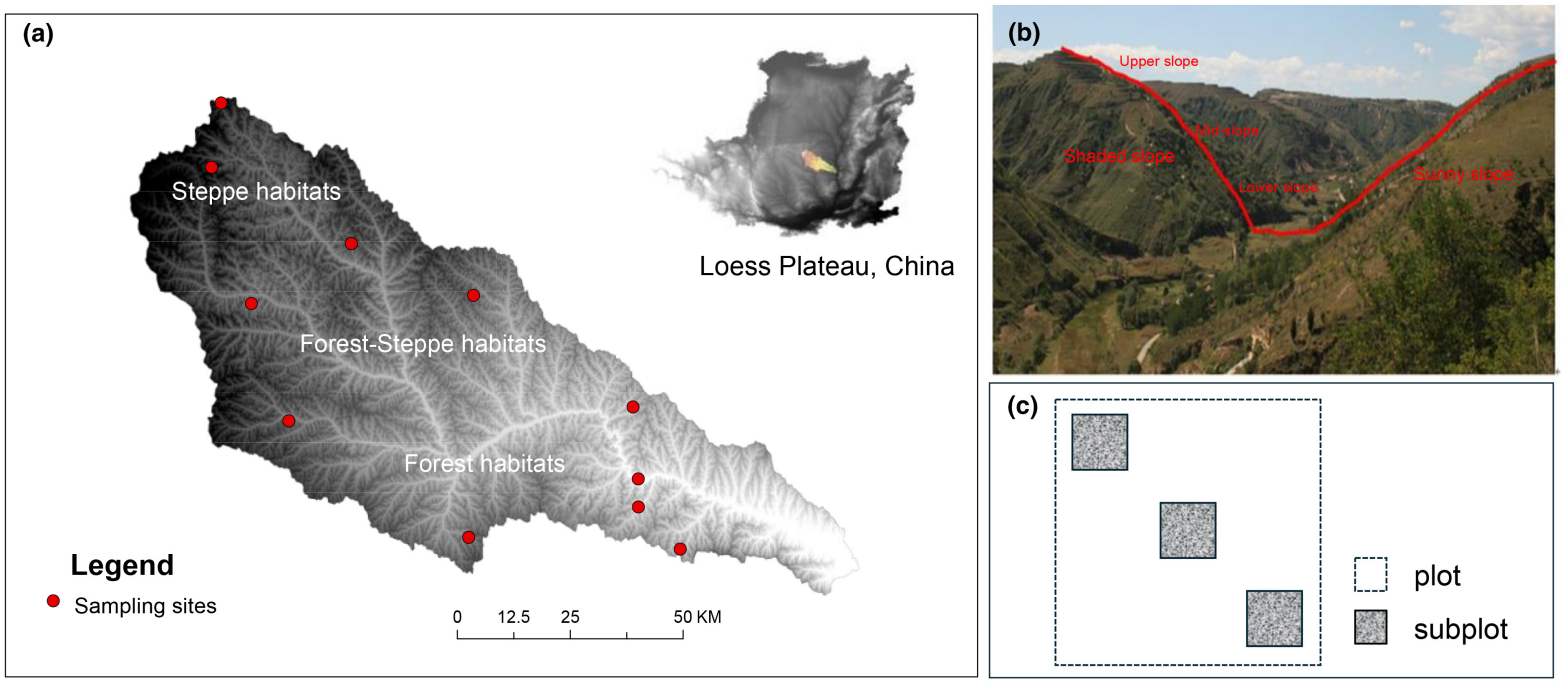
A nested sampling framework was used to test for the signatures of different assembly rules. Utilizing null model techniques, habitat filtering and limiting similarity were examined at two scales (5 × 5 m plots and 1 × 1 m subplots) by contrasting observed trait structure with null expectations. (a) Study area extent, with the site's locations denoted by dots. (b) Small extent: A sample location with six 5 × 5 m plots. (c) Each plot has three subplots. Geographically segregated null models were used to evaluate the spatial partitioning processes between subplots in each plot.

This study was carried out in a typical hilly‐gully region (YRB) of the Loess Plateau, China (36°21′ N–37°29′ N, 108°38′ E–110°29′ E). The basin covers an area of 7725 km^2^. We identified three habitat zones across the study area, namely forest zone, forest‐steppe zone, and steppe zone from southeast to northwest (Figure 1a). YRB had an average altitude and slope of 1216 m and 17°. Forest dominates in the southern portion of YRB, followed by forest‐ steppe in the middle and southeast, and steppe in the northern half. We identified 68 plant species across 11 sites. The most commonly encountered species were *Thymus mongolicus*, *Stipa bungeana*, *Artemisia sacrorum*, *Lespedeza daurica*, *Cleistogenes caespitosa*, *Bothriochloa ischaemum*, and *Artemisia giraldii*.

### 2.2 Data collection

Vegetation surveys were conducted in the peak growing period from June and August 2021. We chose 11 natural vegetation sites to capture the spectrum of the habitat gradients in the region. At each site, a cross‐section line was selected according to a stratified random sampling strategy (using climate, and micro‐terrain) to cover all possible microhabitats resulted from the terrain changes. Six 5 × 5 m plots were set along the cross‐section line, positioned as far as possible from the farmland and roads (Figure 1b). Within each plot, three nested subplots of 1 × 1 m were established (Figure 1c).

### 2.3 Data collection and determination of function trait values

At each plot and subplot, the abundance, cover, and biomass of common species were collected. Overall, 1354 plant individuals from 68 species were surveyed across the sites.

In this study, our functional traits were selected to indicate various life cycle stages of plants. The four traits are plant height (VH), specific leaf area (SLA), leaf dry matter content (LDMC), and leaf nitrogen content (resource utilization strategy, LNC), which were determined according to the standardized method by from 4 to 20 specimens per species, typically 10 (Pérez‐Harguindeguy et al., 2016) (Table 1). We excluded plots with common species coverage less than 80% of the relative vegetation coverage and subsequently omitted three plots to analyze the remaining 60 plots.

**TABLE 1:** Description of the functional traits, both the literature and following the cited protocols.

Trait	Unit	Functions	Reference
Plant height (VH)	cm	Competitive ability, light capture	Westoby et al. (2002)
Specific leaf area (SLA)	mm^2^/mg	Resource acquisition and relative	de Bello et al. (2012)
Leaf Dry Matter Content (LDMC)	mg/mg	Leaf tissue density, stress tolerance	Pérez‐Harguindeguy et al. (2016)
Leaf Nitrogen Content (LNC)		Competitive, photosynthetic performance	(Lebrija‐Trejos et al., 2010), Muller et al. (2011)

As for environmental factors selection and evaluation, we collected the climatic data from the 105 weather stations in the study area and surrounding areas in the past 10 years, and then used the ANUSPLIN interpolation method with altitude as a covariate to interpolate these climatic data into climatic maps (McVicar et al., 2007). During this process, a DEM with a 25 m resolution was used. With these climatic maps, we used the coordinates of each plot to derive the temperature and precipitation as well as the elevation data for statistical analysis.

### 2.4 Statistical analyses

The comparison between observed functional diversity and expectations derived from null models enabled the evaluation of different community assembly rules' influences (Scherrer et al., 2019). Within each habitat zone, we test community assembly rules at plot and subplot levels. The null model and diversity distributions serve as the framework for assembly rules (Cornwell & Ackerly, 2009; Kraft et al., 2008): (a) Trait convergence is suggested when observed diversity at the plot (or subplot) level is lower than null model predictions, indicating habitat filtering influences. (b) Conversely, if observed diversity exceeds null model expectations at either the plot or subplot level, it suggests trait divergence, attributed to limiting similarity as the underlying assembly rule. (c) Spatial partitioning occurs when the β‐component of functional diversity within plots surpasses that of the null model predictions.

FD was quantified using the weighted α‐Rao quadratic entropy index, which accounts for functional trait differences among species relative to their abundance within a community. We then applied the mean pairwise distance (MPD) between species within a quadrat to determine FD for each trait (de Bello et al., 2010). For the β‐component of functional diversity in each plot, the β‐Rao quadratic entropy index was utilized (de Bello et al., 2010).

We considered five different representations of trait diversity: one multidimensional space considering the four growth traits simultaneously (multidimensional space combining all growth traits, CGT), and four one‐dimensional spaces each consisting of one separate growth trait. All traits were log transformed and standardized (Table 1).

It is crucial to create a robust null model for the ecological mechanisms being investigated. Given the associations between species traits (Agrawal, 2020), we treated all traits of each species as a single unit and randomized their allocation by species to simulate the null model sampling. This null model preserved the relative abundance and occurrence frequency of species in each plot/subplot, circumventing unrealistic assumptions. The different assembling processes are determined by the size of the species pool: (a) The species pool with the entire study area as the null model represents habitat filtering; (b) The species pool with the site as the null model represents the limiting similarity. The spatial scale is the grain size, which is divided into 5 m^2^ and 1 m^2^. For each test, whether it is plots or subplots, the null model results are the results of random sampling 999 times. Finally, we calculate the standard effect size (SES) of α‐Rao or β‐Rao using the observed diversity patterns and the null model pattern as follows:

$${\mathrm{SES}}_{\mathrm{Rao}}=\left({\mathrm{Rao}}_{\text{plot}}-\mu_{{\mathrm{Rao}}_{\mathrm{nm}}}\right)/\sigma_{{\mathrm{Rao}}_{\mathrm{nm}}}$$

where ${\mathrm{Rao}}_{\text{plot}}$ is the observed α−/β‐Rao, $\mu_{{\mathrm{Rao}}_{\mathrm{nm}}}$ is the mean of the null model simulation, and $\sigma_{{\mathrm{Rao}}_{\mathrm{nm}}}$ is the α−/β‐Rao simulated by the null model standard deviation. A negative SES of α‐Rao under habitat filtering indicates less functional diversity than the null model, while a positive SES implies greater diversity for limiting similarity. Spatial partitioning within a plot is inferred from a positive SES for β‐Rao. A negative SES of α‐Rao under habitat filtering indicates less functional diversity than the null model, while a positive SES implies greater diversity for limiting similarity. Spatial partitioning within a plot is inferred from a positive SES for β‐Rao.

We assessed the community assembly rules using the Wilcoxon rank‐sum test, comparing the plot‐level α‐Rao with the subplot‐level α‐Rao to determine the assembly impact of spatial scale. To evaluate the significance of spatial partitioning, the Wilcoxon rank‐sum test was also performed to the β‐Rao among subplots. In addition, we fitted the linear mixed‐effects models (LMEs) for SES of growth traits to investigate the influence of environmental factors (mean annual temperature and mean annual precipitation, MAT and MAP) on the strength of community assembling processes, with a random intercept for the plot to control for pseudo replication. Response variables were expressed as a function of MAT, MAP, and all two‐way interactions in our models, different micro‐terrain as random effects. We used Akaike's information criterion (AIC) to select the best model for explaining the community assembly rules. Based on the lowest AIC, the best model was chosen from all combinations of environmental factors. We used the nlme package for R (Team, 2014; Pinheiro et al., 2020) to fit all LMEs with restricted maximum likelihood (REML). We fit the models with maximum likelihood for AIC model comparison (Zuur et al., 2010).

Principal component analyses (PCA) of community‐weighted mean trait values at the subplots level for the three ecotones were carried out (using the R function prcomp). The PCA gives supplementary information of the community assembly rules along the habitat gradients by synthesizing of the differences in community functional traits change among habitats.

## 3 RESULTS

### 3.1 Community assembly rules in relation to spatial scales

**FIGURE 2:**
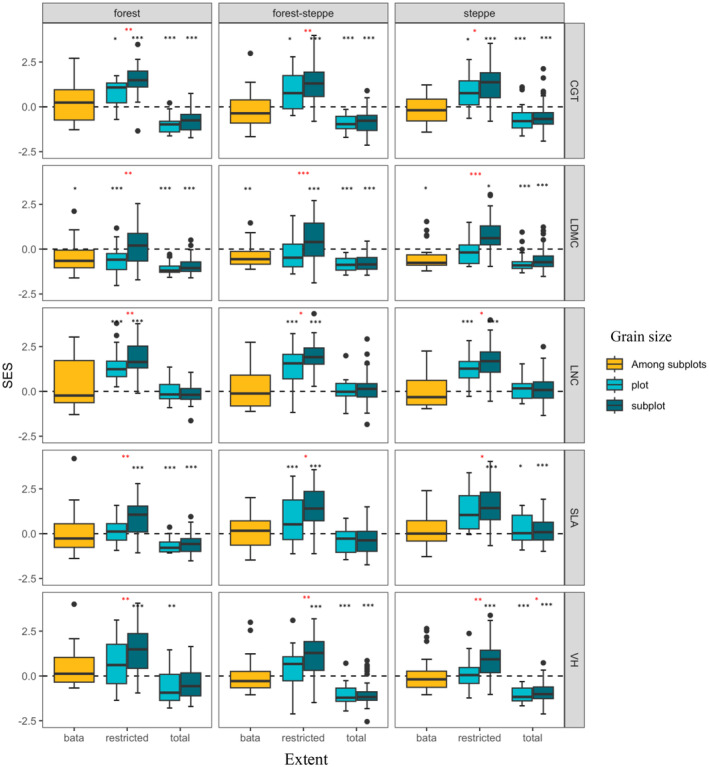
SES' distribution of α‐Rao and β‐Rao within each scale level for extent and grain size. The x‐axis is the spatial scale of grain size for each of the stages of the habitat (steppe, forest‐steppe, and forest). β‐Rao within the plot; restricted, SES of the restricted reshuffling with the species pool in station; total, SES of a‐Rao reshuffling with the species pool in the study area. CGT, combining all growth traits; LDMC, Leaf dry matter content; LNC, Leaf nitrogen content; SLA, Specific leaf area; VH, Vegetation height. Significance symbols above a boxplot mark significant differences from 0, *p < .05; **p < .01; ***p < .001. Red asterisks indicate differences between spatial scales.

Across the three habitat types, the plant community was consistent with the predicted spatial scale. The mean SES of a‐Rao total reshuffling with the species pool in the study area was significantly smaller than the expectation of the null model, and the plot level was smaller than the subplot level, and not significant; the mean SES of the restricted reshuffling with the species pool in site was significantly greater than that of the null model expected at any spatial scale, but the subplot level is significantly higher than the plot level. The mean SES of β‐Rao within the plot did not significantly deviate from envelope of the null model. However, 18%–41% of the plots showed much greater β‐Rao than that expected under assembly. Even so, not all traits have consistent results. In the community assembly patterns reshuffling with the species pool in the study area, no other traits of LNC were significant. From the perspective of traits, the traits of VH and LDMC were more convergent in the community assembly pattern of reshuffling with the species pool in the study area, while the traits of SLA and LNC were more divergent in the assembling mode of restricted reshuffling with the species pool of the station (Figure 2).

**FIGURE 3:**
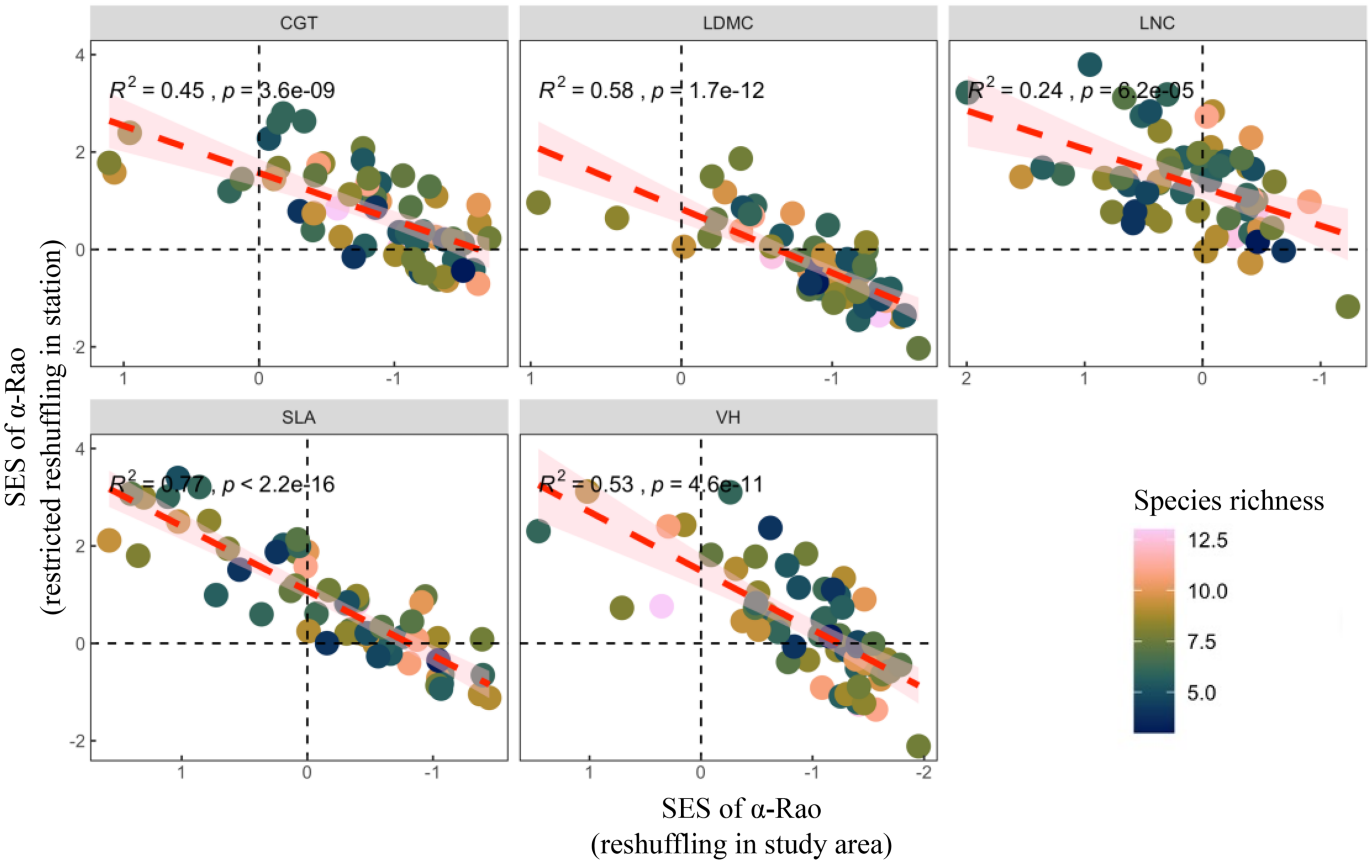
Linear regression model of two extents measured by SES of α‐Rao at the plot level. Different submaps represent different traits, the x‐axis is the reshuffling with the species pool in study area, the y‐ axis is the restricted reshuffling with the species pool in station, and the color spectrum depicts the plots' species richness from 3 to 13. The x‐axis is reversed.

**FIGURE 4:**
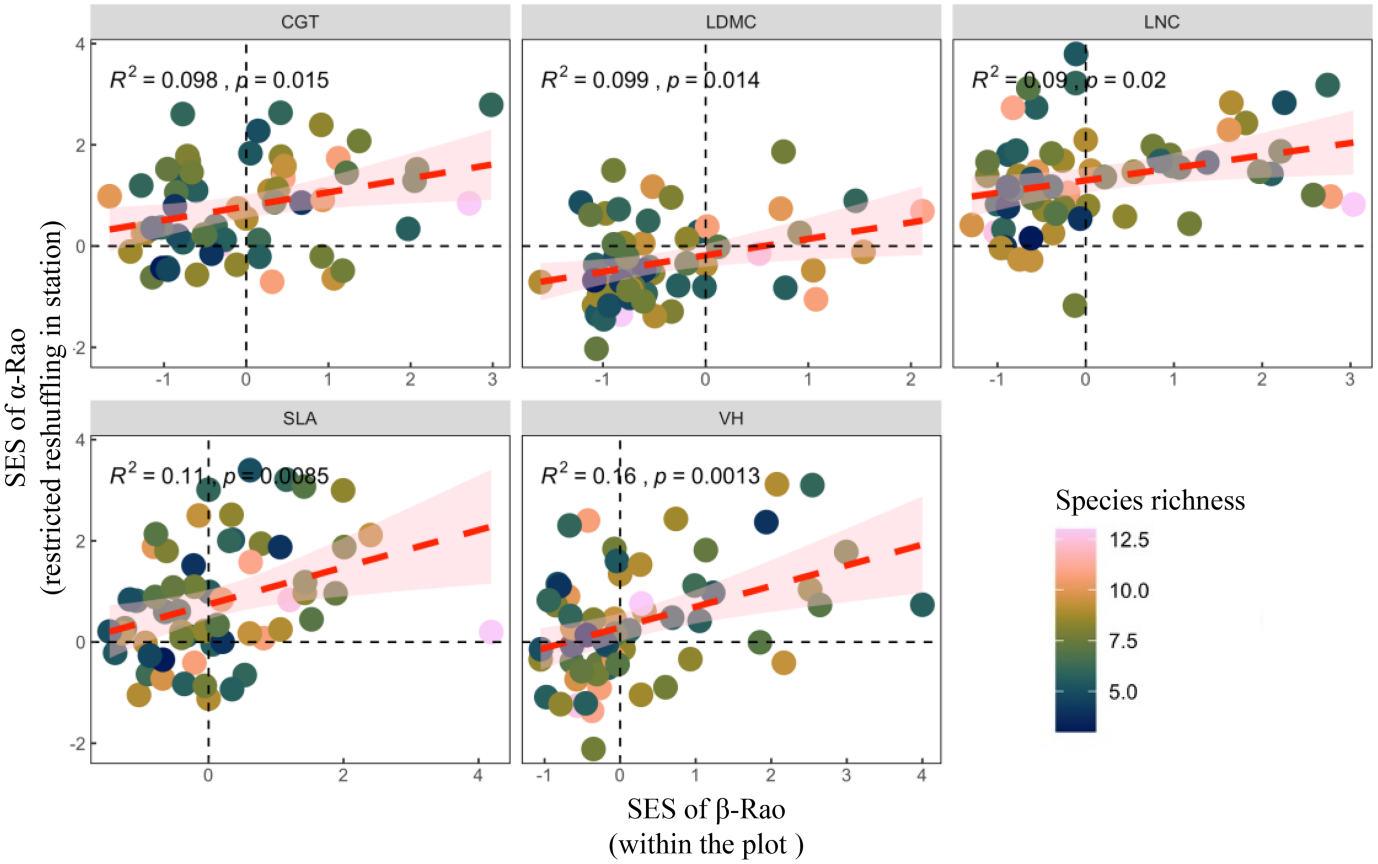
Linear regression model of SES of α‐Rao at the plot level and SES of β‐Rao. Different submaps represent different traits, the x‐axis is SES of β‐Rao within plot (among subplots), and the y‐ axis is SES of α‐Rao of restricted reshuffling with the species pool in site.

There is also an association between community assembly patterns despite our independent null tests for community assembly patterns. We submitted linear regression model on the mean SES of a‐Rao reshuffling with the species pool in the study area and the mean SES of the restricted reshuffling with the species pool in site at the plot level, and the results showed that the two were negatively correlated (*R* ^2^ ≈ .53; Figure 3). Furthermore, the mean SES of β‐Rao within the plot is positively correlated with the mean SES of the restricted reshuffling with the species pool in site (*R* ^2^ ≈ .075; Figure 4).

### 3.2 Combining effects of MAT and MAP on community assembly patterns

Using a linear mixed‐effects model that includes MAP, MAT, and their interactions, this study investigates the regression relationship between community assembly rules and environmental factors (Table S1). Models 2 and 4 with temperature gradient are the best models for habitat filtering, with the lowest AIC (Table 2). Model 4 is the best model for limiting similarity because it has the lowest AIC. These findings suggest that one important environmental factor that sets apart the community assembly process is temperature. The optimal LMEs revealed that the habitat filtering effect was stronger in some habitats of YRB with low temperature (*p <* .05). The limiting similarity effect was stronger (*p <* .05) with higher temperature (Table 2). Furthermore, in this study, fixed‐effect environmental factors and random effects were more significant in explaining environmental filtering than limiting similarity.

**TABLE 2:** The linear mixed models investigating the effects of environment factors on community assembly rules for each trait.

Community assembly rules	Model	Intercept	MAT	MAP	MAT*rainfall	*R* ^2^ marginal	*R* ^2^ conditional
CGT‐a	2	**70.42**	**−6.71**	**−0.13**	**0.01**	.33	.729
SLA‐a	2	**64.88**	**−5.98**	**−0.12**	**0.01**	.312	.686
VH‐a	4	**−3.52**	**0.26**			.14	.201
LNC‐a	4	**1.48**	**−0.14**			.051	.12
LDMC‐a	4	**0.37**	**−0.13**			.068	.122
CGT‐b	2	**40.94**	−3.76		**0.01**	.113	**.217**
SLA‐b	4	**4.8**	−0.4			.098	.369
VH‐b	4	**−4.06**	**0.45**			.146	.185
LNC‐b	4	−1.36	**0.27**			.076	.145
LDMC‐b	1	**−3.1**	−0.06	0.01		.058	.13
CGT‐c	null	0.01				0	
SLA‐c	null	0.15				0	
VH‐c	null	0.23				0	
LNC‐c	null	0.2				0	
LDMC‐c	3	**−3.08**	0.01			.057	.1
CGT‐d	2	**33.76**	**−3.2**	**−0.07**	**0.01**	.076	.428
SLA‐d	2	**36.18**	**−3.47**	**−0.07**	**0.01**	.088	.409
VH‐d	4	**−3.24**	**0.24**			.109	.251
LNC‐d	4	**1.19**	**−0.11**			.025	.081
LDMC‐d	2	**23.57**	**−2.23**	**−0.05**	0	.096	.322
CGT‐e	2	29.17	−2.54	−0.06	0.01	.057	.142
SLA‐e	null	**1.33**					.14
VH‐e	1	0.76	**0.53**	**−0.01**		.103	.192
LNC‐e	null	**1.75**					.017
LDMC‐e	1	−0.65	**−0.3**	**0.01**		.05	.1

*Note*: Mean annual precipitation (MAP) and mean annual temperature (MAT) were included in the models as environment factors. Significant results are shown in bold and indicated (*p* < .05). a: SES of α‐Rao plot level (reshuffling with the species pool in the study area), b: SES of α‐Rao plot level (restricted reshuffling with the species pool in the study area), c: SES of β‐Rao (within plot), d: SES of α‐Rao subplot level (reshuffling with the species pool in the study area), e: SES of α‐Rao subplot level (restricted reshuffling with the species pool in the study area). *R* ^2^ marginal, amount of variation that is explained by fixed factors (covariates); R^2^conditional, amount of variation that is explained by both fixed and random factors.

## 4 DISCUSSION

Our results provide experimental evidence that spatial scales and environmental gradients can be used to effectively separate the presence of distinct assembly rules. In contrast with recent trait‐based community analyses conducted in more mesic ecosystems, much of the SES value identified in our study were placed outside the null model envelopes with a reasonably high frequency (Freschet et al., 2011; Schrader et al., 2021). Overall, we discovered that determining the working signature of the underlying mechanisms guiding community assembly requires consideration of spatial scale. At the plot level, habitat filtering is more important, while at the smaller level of subplots, limiting similarity is more important. This finding suggests that non‐random assembly rules strongly influence the structure of communities. Most of the plots are in the first quadrant as shown in Figure 3, where habitat filtering and limiting similarity are the dominant patterns of community assembly. Moreover, further analyses relating the observed community process to its environmental factors allow us to generalize assembly rules from one habitat to another, it also provides a framework for managing of degraded ecosystems.

### 4.1 The spatial scale and trait selection affect community assembly patterns

We detected the evidence of habitat filtering and limiting similarity, which consistent with the previous research (Gotzenberger et al., 2012; Holdaway & Sparrow, 2006; Scherrer et al., 2019; Zheng et al., 2022). Nonetheless, the findings of this study show that limiting similarity is stronger at the subplot level than at the plot level, and habitat filtering is stronger at the plot level, but not significant compared with the subplot in the YRB. Furthermore, we typically find divergence or convergence but did not for all the analyzed traits. It is also possible to be report some traits convergence and the other divergence within the same system (Cavender‐Bares et al., 2004; Conti et al., 2017), such as, the SES of the restricted reshuffling with the species pool in site at the plot level in YRB (Figure 2).

The prevalence of habitat filtering confirms what was observed: Plant community assembly is shaped by environmental conditions via functional traits. The habitat filtering for traits within a threshold suited to survival and growth under specific conditions (López‐Angulo et al., 2020). Consequently, sparser grassland habitats typically foster a more conservative assemblage of plants adapted to harsh conditions such as wind, cold, and low nutrient availability, whereas more intricate habitats support a broader spectrum of species that utilize diverse resource acquisition strategies (Choler, 2005; Takahashi & Tanaka, 2016).This hypothesis is supported by the PCA results of the observed community‐weight means trait values (Figure S1).

Limiting similarity is a pivotal role at the subplot level (1m^2^), where competition for resources is intensified compared to the plot level (Schwilk & Ackerly, 2005). Our findings suggest that limiting similarity influences grassland communities not only at a finer scale but also at the broader plot scale within arid regions, as indicated by the observed trait divergence. Moreover, spatial partitioning may underlie this divergence, with trait divergence at the plot level possibly arising from the spatial separation of species' niches. This relationship is underscored by a positive correlation between limiting similarity and spatial partitioning at the plot scale (Figure 4).

Fine‐scale spatial partitioning is a relatively infrequent deterministic community assembly patterns in the YRB of Loess plateau, even though the observed positive association between spatial partitioning and limiting similarity at the plot‐level as shown in Figure 4. The findings of large‐scale community assembling process inference should be challenged. The observed spatial partitioning in plant community assembly indicates that fine‐scale correlation analysis will be important in future studies (Laliberte et al., 2013), potentially employing β‐diversity patterns to describe spatially explicit texts (Siefert, 2012) and fine‐scale physical environmental (Stark et al., 2017).

Regarding trait selection, despite minor discrepancies such as those between LNC and LDMC, the overall trend of community assembly process remains consistent spatial scales and environmental changes. While integrating traits into a unified functional space is a research direction in functional diversity (Koffel et al., 2022; Laughlin, 2014; Maharjan et al., 2021), underlying significant mechanisms may be obscured if this method is used to analyze trait‐based community assembly without consideration (Conti et al., 2017). It is also possible that unexamined traits in our study could reveal more pronounced deterministic or stochastic patterns, even though we selected four growth traits for as many individuals as possible.

Although null model are widely used in community assembly researches, they are observational in nature and do not imply causation (Marteinsdottir et al., 2018), akin to other empirical methods (Marteinsdottir et al., 2018; Mugnai et al., 2022). Comparisons to null models reveal that trait divergence is influenced by limiting similarity, while convergence is driven by habitat filtering. These conclusion simplifies the assembly process while ignoring important ecological processes (HilleRisLambers et al., 2012; Munkemuller et al., 2020). It is argued that while two opposing forces may generate random patterns within an ecosystem assembly (Cornwell & Ackerly, 2009; Gotzenberger et al., 2016; Maire et al., 2012), deviations of standard effect size (SES) value distributions from zero can hint at the prevailing processes affecting a particular trait at a specific scale. Additionally, trait convergence might result from different mechanisms, such as habitat filtering and competitive exclusion (Mayfield & Levine, 2010), and our findings that the plot‐level trait convergence is more pronounced than at the subplot level suggest the presence of habitat filtering. This study further investigates concurrent mechanisms of habitat filtering and limiting similarity by utilizing different species pools for randomized tests of trait diversity, offering clues to where these processes may overlap.

We support the findings of Munkemuller et al. (2012) that inferring assembly rules from diversity patterns is more relevant when based on multiple criteria rather than a singular metric. Combining taxonomic, functional, and phylogenetic diversity measures using α‐ and β‐indices by Munkemuller et al. (2012) and Hardy (2008). Many biodiversity facets along environmental gradients and spatial scales, according to Mugnai et al. (2022), allow for the advancement of both theoretical and practical elements of community assembly. Our work highlights the importance of integrating nested scale assembling rule testing with the definition of diverse species pools.

### 4.2 Variation in the assembly rules was influenced by environmental factors

Our study discovered that environmental factors play a significant role in the community assembly rules as presented in Table 2. This supports the claim that local environmental conditions exert assembly influence on certain aspects of the assembly rule and community structure (LaManna et al., 2021; Wang et al., 2022). MAT restricted the range of viable strategies by favoring a specific set of traits (de Bello et al., 2006), a pattern not as evident across MAP gradient (Table 2). This could be because trait convergence patterns are more pronounced within certain functional types of plants, rather than across all hierarchical levels of species. In the YRB, the southeast region, which enjoys higher precipitation and optimal temperatures, exhibits lower herbaceous community richness and a predominance of forest communities (forest zones). Species distributions are influenced by their climatic preferences, which in turn shape community composition (Banares‐de‐Dios et al., 2020). The physical conditions that dictate microclimate development within forest belts play a crucial role in structuring and functioning grassland communities (Haesen et al., 2021; Hakkenberg et al., 2020). De Frenne et al. (2019) claim that the observed patterns are more a result of microhabitat conditions than direct influences from the broader physical environment. Therefore, tree canopies may lessen the impact of warming on the biodiversity and functionality of forests. Additionally, larger life‐form trees may exclude stronger competitors from the habitat, implying that individuals' interactions are hierarchically influenced (Kunstler et al., 2012; Mudrak et al., 2016). These insights demonstrated that changes in environmental conditions do not uniformly relieve all plants from resource restrictions, especially for herbaceous communities. The resource competition engendered by shifts in vegetation type may be overlooked in studies focused on a single vegetation type (Peralta et al., 2019).

The link between community assembly rules and environmental factors was inconsistent across different traits, suggesting that environmental shifts act on traits rather than on individual organisms. Moreover, individual resource allocation facilitates the functional balance of each plant through intraspecific and interspecific trait trade‐offs and coordination in specific environment (Anderegg et al., 2018; Shipley et al., 2006). Corroborated by field data and principal component analysis (PCA), we found that MAT is a significant environmental factor affecting the community assembly rule (Figure S1).

Our analysis revealed only a weak correlation between the signals of limiting similarity and environmental factors. The impact of limiting similarity on different traits varied in response to environmental factors (Table 2). As argued by Maire et al. (2012), habitat filtering and limiting similarity occur concurrently within communities along the habitat gradient. VH and LNC are positively correlated related to MAT, suggesting an increase in the influence of limiting similarity as the MAT rises from low to high. This result is apparently contradictory to the stress gradient hypothesis, which predicts on increase in limiting similarity in productive lowlands in which plant competition is expected to be more intense (Chalmandrier et al., 2017; He et al., 2013). However, LDMC and SLA showed inconsistent responses, possibly due to water scarcity and the diversity of microhabitats within the understory of plots in the YRB (Luzuriaga et al., 2018; Scherrer et al., 2019). In conclusion, multiple trade‐offs in different dimensions shape the community assembly in the YRB.

Habitat filtering seemed to decrease as limiting similarity rose along MAT gradient (Table 2). However, no significant trend was detected in community assembly rules along MAP gradient. This would indicate that the trait selection dimensions considered are not entirely aligned with the MAP gradient. Furthermore, in the context of degraded ecosystems, vegetation restoration significantly alters the original vegetation community structure, with alien species previously shown to diminish the biodiversity of native plants (Guo et al., 2021). Consequently, a comprehensive understanding of grassland assembly rules should incorporate aspects of land management within the YRB. These insights underscore the complexity of community assembly, where variable responses to individual traits necessitate consideration of multiple trait dimensions.

## 5 CONCLUSIONS

**FIGURE 5:**
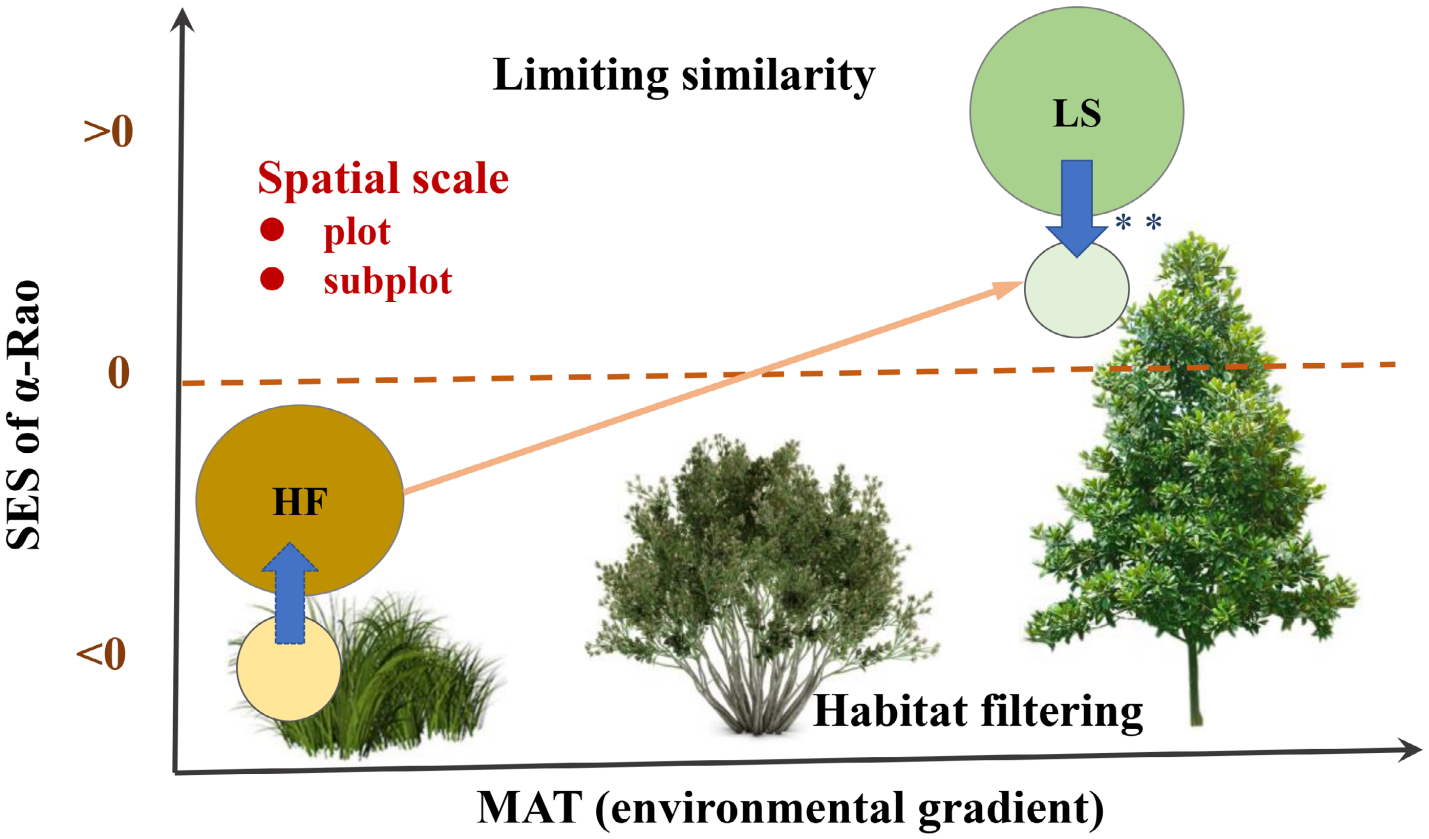
A conceptual framework describing the conclusions shows how spatial scales and environmental factors affect the balance of grassland community assembly rules (HF, habitat filtering; LS, limiting similarity). Orange arrows indicate the result of habitat conversion. Blue arrows indicate spatial scale effects. Larger circles indicate the plot level. Smaller circles indicate the subplot level. “*” indicates significant test.

Null models based on different constraints can disentangle the roles of habitat filtering and limiting similarity in community assembly rules. The dominating assembly rule at the coarse scale across YRB was habitat filtering, particularly for LDMC, selecting for plants with low LDMC at warm habitats and high LDMC at cold habitats. Limiting similarity was detected at plot and subplot levels, particularly for light‐acquisition traits (VH), but was stronger and dominating at the subplot level (Figure 5). Plot‐level trait divergence presumably indicating spatial partitioning rather than limiting similarity as the underlying mechanism, as the two mechanisms are related. Our study suggests that detecting community assembly rules requires considering spatial partitioning and high‐resolution, multidimensional abiotic data to understand species coexistence and predict changes in biodiversity. Altogether, our study emphasizes the importance of spatial scales in decoding community assembly rules and illustrates the mechanisms of biodiversity maintaining in plant communities by combining environmental gradient.

## AUTHOR CONTRIBUTIONS

**Cheng Zheng:** Conceptualization (equal). **Haijing Shi:** Conceptualization (supporting); formal analysis (equal); writing – original draft (supporting); writing – review and editing (supporting). **Jiaqi Wei:** Investigation (equal); methodology (equal). **Mengying Cui:** Investigation (supporting); methodology (supporting). **Ziqi Lin:** Investigation (supporting); methodology (supporting). **Yuan Gao:** Investigation (lead); methodology (supporting). **Liuhuan Yuan:** Methodology (supporting). **Zhongming Wen:** Conceptualization (equal); funding acquisition (equal); resources (equal); supervision (equal).

## CONFLICT OF INTEREST STATEMENT

The authors declare that they have no known competing financial interests or personal relationships that could have appeared to influence the work reported in this paper.

## Supporting information


Data S1.

Data S2.

Data S3.

Figure S1.
Table S1.

## Data Availability

The data supporting the results used to generate the analyses are available at the Supplementary materials.
