# Supplementary material for: Evidence that spatial scale and environment factors explain grassland community assembly in woodland–grassland ecotones
Source: Ecol Evol. 2024 Jul 3;14(7):e11644. doi: 10.1002/ece3.11644 (PMC11221066; doi:10.1002/ece3.11644)
Supplement: Supplementary file 4 — Figure S1. Table S1. [file ECE3-14-e11644-s002.docx]

**Title: Evidence that spatial scale and environment factors explains community assembly in grassland communities**

**TABLE S1**. Proposed linear mixed models to explain community assembly processes and their respective AIC values at both sites. Best fit model is represented in bold.

| Community assembly process | model | | Model structure | AIC | Community assembly process | model | | Model structure | AIC |
| --- | --- | --- | --- | --- | --- | --- | --- | --- | --- |
| CGT-a | | null | 1+(1\|site) | 121.78 | CGT-d | | null | 1+(1\|site) | 324.02 |
| CGT-a | | 1 | MAP+MAT+(1\|site) | 124.66 | CGT-d | | 1 | MAP+MAT+(1\|site) | 473.09 |
| CGT-a | | 2 | MAP*MAT+(1\|site) | **120.35** | CGT-d | | 2 | MAP*MAT+(1\|site) | **323.25** |
| CGT-a | | 3 | MAP+(1\|site) | 123.41 | CGT-d | | 3 | MAP+(1\|site) | 324.92 |
| CGT-a | | 4 | MAT+(1\|site) | 122.47 | CGT-d | | 4 | MAT+(1\|site) | 32575 |
| SLA-a | | null | 1+(1\|site) | 140.02 | SLA-d | | null | 1+(1\|site) | 371.45 |
| SLA-a | | 1 | MAP+MAT+(1\|site) | 137.59 | SLA-d | | 1 | MAP+MAT+(1\|site) | 373.93 |
| SLA-a | | 2 | MAP*MAT+(1\|site) | **134.07** | SLA-d | | 2 | MAP*MAT+(1\|site) | **371.07** |
| SLA-a | | 3 | MAP+(1\|site) | 139.19 | SLA-d | | 3 | MAP+(1\|site) | 372.45 |
| SLA-a | | 4 | MAT+(1\|site) | 135.63 | SLA-d | | 4 | MAT+(1\|site) | 371.99 |
| VH-a | | null | 1+(1\|site) | 125.94 | VH-d | | null | 1+(1\|site) | 349.62 |
| VH-a | | 1 | MAP+MAT+(1\|site) | 124.24 | VH-d | | 1 | MAP+MAT+(1\|site) | 347.45 |
| VH-a | | 2 | MAP*MAT+(1\|site) | 126.24 | VH-d | | 2 | MAP*MAT+(1\|site) | 349.44 |
| VH-a | | 3 | MAP+(1\|site) | 126.41 | VH-d | | 3 | MAP+(1\|site) | 350.66 |
| VH-a | | 4 | MAT+(1\|site) | **122.44** | VH-d | | 4 | MAT+(1\|site) | **345.89** |
| LNC-a | | null | 1+(1\|site) | 115.35 | LNC-d | | null | 1+(1\|site) | 368.58 |
| LNC-a | | 1 | MAP+MAT+(1\|site) | 115.93 | LNC-d | | 1 | MAP+MAT+(1\|site) | 370.51 |
| LNC-a | | 2 | MAP*MAT+(1\|site) | 117.77 | LNC-d | | 2 | MAP*MAT+(1\|site) | 372.46 |
| LNC-a | | 3 | MAP+(1\|site) | 114.81 | LNC-d | | 3 | MAP+(1\|site) | 369.71 |
| LNC-a | | 4 | MAT+(1\|site) | **114.08** | LNC-d | | 4 | MAT+(1\|site) | **368.5** |
| LDMC-a | | null | 1+(1\|site) | 115.35 | LDMC-d | | null | 1+(1\|site) | 248.41 |
| LDMC-a | | 1 | MAP+MAT+(1\|site) | 115.93 | LDMC-d | | 1 | MAP+MAT+(1\|site) | 249.68 |
| LDMC-a | | 2 | MAP*MAT+(1\|site) | 117.77 | LDMC-d | | 2 | MAP*MAT+(1\|site) | **246.68** |
| LDMC-a | | 3 | MAP+(1\|site) | 114.81 | LDMC-d | | 3 | MAP+(1\|site) | 247.91 |
| LDMC-a | | 4 | MAT+(1\|site) | **114.14** | LDMC-d | | 4 | MAT+(1\|site) | 248.24 |
| CGT-b | | null | 1+(1\|site) | 165.38 | CGT-e | | null | 1+(1\|site) | 471.29 |
| CGT-b | | 1 | MAP+MAT+(1\|site) | 166.49 | CGT-e | | 1 | MAP+MAT+(1\|site) | 473.09 |
| CGT-b | | 2 | MAP*MAT+(1\|site) | **163.3** | CGT-e | | 2 | MAP*MAT+(1\|site) | **471.4** |
| CGT-b | | 3 | MAP+(1\|site) | 166.11 | CGT-e | | 3 | MAP+(1\|site) | 473.05 |
| CGT-b | | 4 | MAT+(1\|site) | 164.5 | CGT-e | | 4 | MAT+(1\|site) | 471.39 |
| SLA-b | | null | 1+(1\|site) | 190.26 | SLA-e | | null | 1+(1\|site) | **516.72** |
| SLA-b | | 1 | MAP+MAT+(1\|site) | 191.54 | SLA-e | | 1 | MAP+MAT+(1\|site) | 518.78 |
| SLA-b | | 2 | MAP*MAT+(1\|site) | 190.65 | SLA-e | | 2 | MAP*MAT+(1\|site) | 518.21 |
| SLA-b | | 3 | MAP+(1\|site) | 190.89 | SLA-e | | 3 | MAP+(1\|site) | 518.58 |
| SLA-b | | 4 | MAT+(1\|site) | **189.57** | SLA-e | | 4 | MAT+(1\|site) | 517.38 |
| VH-b | | null | 1+(1\|site) | 188.8 | VH-e | | null | 1+(1\|site) | 523.99 |
| VH-b | | 1 | MAP+MAT+(1\|site) | 183.96 | VH-e | | 1 | MAP+MAT+(1\|site) | **519.82** |
| VH-b | | 2 | MAP*MAT+(1\|site) | 184.78 | VH-e | | 2 | MAP*MAT+(1\|site) | 520.01 |
| VH-b | | 3 | MAP+(1\|site) | 190.18 | VH-e | | 3 | MAP+(1\|site) | 525.96 |
| VH-b | | 4 | MAT+(1\|site) | **122.44** | VH-e | | 4 | MAT+(1\|site) | 522.18 |
| LNC-b | | null | 1+(1\|site) | 171.47 | LNC-e | | null | 1+(1\|site) | **455.34** |
| LNC-b | | 1 | MAP+MAT+(1\|site) | 170.22 | LNC-e | | 1 | MAP+MAT+(1\|site) | 457.2 |
| LNC-b | | 2 | MAP*MAT+(1\|site) | 172.22 | LNC-e | | 2 | MAP*MAT+(1\|site) | 459.2 |
| LNC-b | | 3 | MAP+(1\|site) | 172.2 | LNC-e | | 3 | MAP+(1\|site) | 457.04 |
| LNC-b | | 4 | MAT+(1\|site) | **168.62** | LNC-e | | 4 | MAT+(1\|site) | 455.52 |
| LDMC-b | | null | 1+(1\|site) | 148.8 | LDMC-e | | null | 1+(1\|site) | 498.67 |
| LDMC-b | | 1 | MAP+MAT+(1\|site) | **84.057** | LDMC-e | | 1 | MAP+MAT+(1\|site) | **494.4** |
| LDMC-b | | 2 | MAP*MAT+(1\|site) | 146.957 | LDMC-e | | 2 | MAP*MAT+(1\|site) | 495.89 |
| LDMC-b | | 3 | MAP+(1\|site) | 84.476 | LDMC-e | | 3 | MAP+(1\|site) | 499.77 |
| LDMC-b | | 4 | MAT+(1\|site) | 145.781 | LDMC-e | | 4 | MAT+(1\|site) | 499.61 |
| CGT-c | | null | 1+(1\|site) | **180.61** |  | |  |  |  |
| CGT-c | | 1 | MAP+MAT+(1\|site) | 181.96 |  | |  |  |  |
| CGT-c | | 2 | MAP*MAT+(1\|site) | 181.85 |  | |  |  |  |
| CGT-c | | 3 | MAP+(1\|site) | 182.31 |  | |  |  |  |
| CGT-c | | 4 | MAT+(1\|site) | 182.17 |  | |  |  |  |
| SLA-c | | null | 1+(1\|site) | **178.01** |  | |  |  |  |
| SLA-c | | 1 | MAP+MAT+(1\|site) | 187.86 |  | |  |  |  |
| SLA-c | | 2 | MAP*MAT+(1\|site) | 186.82 |  | |  |  |  |
| SLA-c | | 3 | MAP+(1\|site) | 185.86 |  | |  |  |  |
| SLA-c | | 4 | MAT+(1\|site) | 185.93 |  | |  |  |  |
| VH-c | | null | 1+(1\|site) | **191.2** |  | |  |  |  |
| VH-c | | 1 | MAP+MAT+(1\|site) | 192.92 |  | |  |  |  |
| VH-c | | 2 | MAP*MAT+(1\|site) | 193.3 |  | |  |  |  |
| VH-c | | 3 | MAP+(1\|site) | 191.3 |  | |  |  |  |
| VH-c | | 4 | MAT+(1\|site) | 192.93 |  | |  |  |  |
| LNC-c | | null | 1+(1\|site) | **195.22** |  | |  |  |  |
| LNC-c | | 1 | MAP+MAT+(1\|site) | 196.31 |  | |  |  |  |
| LNC-c | | 2 | MAP*MAT+(1\|site) | 198.22 |  | |  |  |  |
| LNC-c | | 3 | MAP+(1\|site) | 196.87 |  | |  |  |  |
| LNC-c | | 4 | MAT+(1\|site) | 196.75 |  | |  |  |  |
| LDMC-c | | null | 1+(1\|site) | 144.91 |  | |  |  |  |
| LDMC-c | | 1 | MAP+MAT+(1\|site) | 145.19 |  | |  |  |  |
| LDMC-c | | 2 | MAP*MAT+(1\|site) | 146.96 |  | |  |  |  |
| LDMC-c | | 3 | MAP+(1\|site) | **143.35** |  | |  |  |  |
| LDMC-c | | 4 | MAT+(1\|site) | 145.78 |  | |  |  |  |

**Note: mean annual precipitation (MAP), mean annual temperature (MAT) were included in the models as environment factors. Significant results are shown in bold and indicated(p<0.05). a:SES of α-Rao plot level (reshuffling with the species pool in the study area), b: SES of α-Rao plot level (restricted reshuffling with the species pool in the study area), c: SES of β-Rao (within plot), d: SES of α-Rao subplot level (reshuffling with the species pool in the study area), e: SES of α-Rao subplot level (restricted reshuffling with the species pool in the study area). The best models with lowest AIC were shown in bold and indicated.**


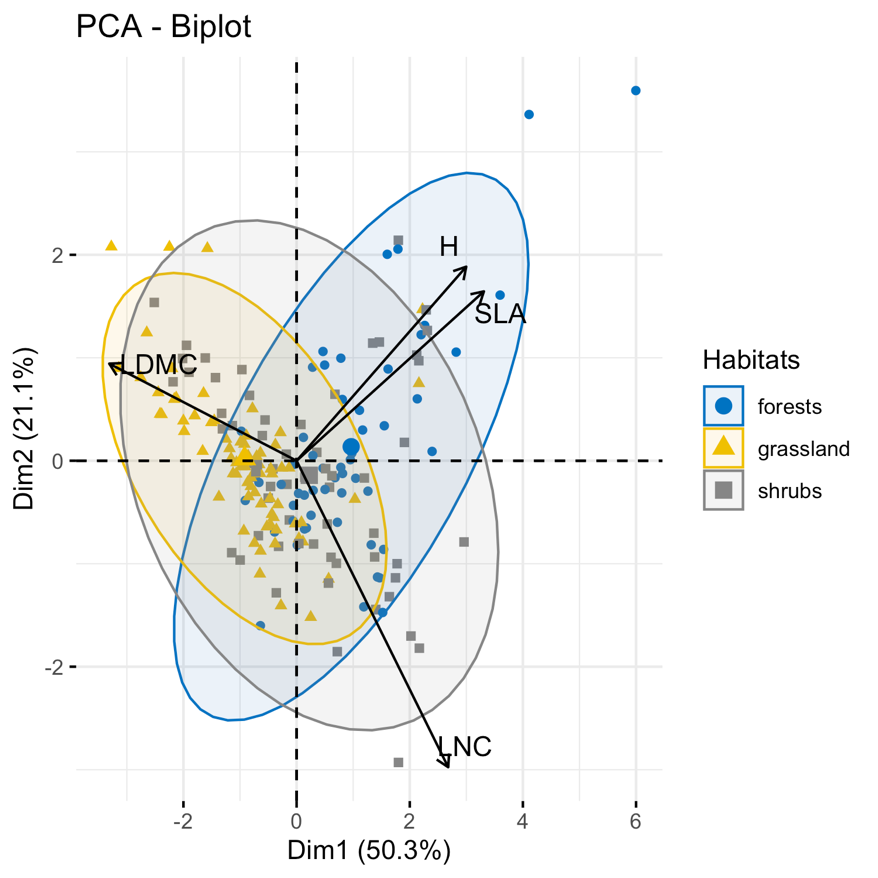


**FIGURE S1.** Principal components analysis (PCA) of species composition in individual subplot with community- weighted means of four studied traits displayed as supplementary variables. The functional traits used in the PCA are plant height (m), leaf dry matter content (LDMC, mg|g), Leaf Nitrogen Content (LNC, mg|g) and specific leaf area (SLA, mm2|mg). The first two axes explain 71.4% of data variation
